# Quantitative analysis of paravalvular leak of transcatheter aortic valves using cardiac MR

**DOI:** 10.1186/1532-429X-15-S1-P272

**Published:** 2013-01-30

**Authors:** Gareth Crouch, Jayme Bennetts, Ajay Sinhal, Craig Bradbrook, Suchi Grover, Carmine DePasquale, Majo Joseph, Joseph Selvanayagam

**Affiliations:** 1 Cardiovascular Medicine, Flinders University, Bedford Park, SA, Australia; 2 Cardiothoracic Surgery, Flinders Medical Centre, Adelaide, SA, Australia; 3 Centre for Cardiovascular Magnetic Resonance Research, Flinders University, Adelaide, SA, Australia

## Background

There is now extensive registry and clinical trial data demonstrating an increased incidence of paravalvular leak following transcatheter aortic valve implantation (TAVI) when compared with open aortic valve replacement (AVR). Despite recent improvements in both hardware and software, echocardiographic measurement of aortic regurgitation (AR) largely remains qualitative in nature. Cardiovascular magnetic resonance (CMR) is able to directly quantify AR with high accuracy and reproducibility by using the technique of phase-contrast velocity mapping. We sought to compare CMR quantitative analysis of AR with concurrently collected echocardiographic measurements in patients undergoing both TAVI and open AVR.

## Methods

Thirty-eight patients (20 male) with confirmed severe aortic stenosis undergoing either TAVI (20 patients) or high risk (euroSCORE >12) open AVR. CMR (1.5T Siemens Aera) and transthoracic echocardiography (TTE, General Electric Vivid E9) were carried out pre-operatively and within two weeks post-operatively. Both CMR and echo were performed on the same day (consecutively) and in random order and analysed by separate blinded operators. CMR protocol consisted of standard LV short and long axis views (SSFP images) and forward and regurgitant aortic flows using through-plane phase-contrast velocity mapping (free breathing, retrospective gating). The image plane was placed ≈0.5 cm above the aortic valve at end-diastole, but a position in the aortic root was maintained throughout the cardiac cycle. Mild, moderate and severe AR by CMR was defined as regurgitation fraction of mild ≤15%, moderate 16-25%, moderate-severe 26-48%, and severe >48% [1].

## Results

Mean ages and log euroSCORE's were similar. Post-operative CMR and TTE were conducted at a median of 6.0 days for TAVI and 7.3 days for Open. Mean preoperative left ventricular (LV) and right ventricular (RV) ejection fractions (EF) were similar in the 2 groups using CMR. Post-operative LVEF was also similar in both groups. Post-procedure regurgitant fraction using CMR was higher in the transcatheter group when compared to the open AVR group (17.7 vs. 4.8% p<0.01). Using published criteria for classification of severity [1], regurgitation severity was estimated at significantly lower grading by echo than CMR (p<0.01 see table below).

**Table 1:** 

OPEN	None/Trivial	Mild	Moderate	Moderate-Severe	Severe
CMR	84.6%	15.4%	0%	0%	0%
Echo	92.3%	7.7%	0%	0%	0%

TAVI					

CMR	15%	35%	20%	20%	10%
Echo	35%	35%	25%	5%	0%

## Conclusions

When compared to CMR based quantitative analysis, TTE consistently underestimated the degree of paravalvular aortic regurgitation, likely due to image degradation associated with the implanted valve and/or poor echocardiographic windows. This underestimation may in part explain the recent findings of the PARTNER trial, which showed that ‘mild' paravalvular leak (as defined by TTE) was a predictor of medium-term mortality.

## Funding

Research grant from Flinders Cardiothoracic Research Fund
